# Disability, Health, and Multiple Chronic Conditions Among People Eligible for Both Medicare and Medicaid, 2005–2010

**DOI:** 10.5888/pcd10.130064

**Published:** 2013-09-19

**Authors:** Michael H. Fox, Amanda Reichard

**Affiliations:** Author Affiliations: Amanda Reichard, University of Kansas, Lawrence, Kansas.

## Abstract

**Introduction:**

People who are eligible for both Medicare and Medicaid (dual eligibles) and who have disabilities and multiple chronic conditions (MCC) present challenges for treatment, preventive services, and cost-effective access to care within the US health system. We sought to better understand dual eligibles and their association with MCC, accounting for sociodemographic factors inclusive of functional disability category.

**Methods:**

Medical Expenditure Panel Survey (MEPS) data for 2005 through 2010 were stratified by ages 18 to 64 and 65 or older to account for unique subsets of dual eligibles. Prevalence of MCC was calculated for those with physical disabilities, physical plus cognitive disabilities, and all others, accounting for sociodemographic and health-related factors. Adjusted odds for having MCC were calculated by using logistic regression.

**Results:**

Of dual eligibles aged 18 to 64, 53% had MCC compared with 73.5% of those aged 65 or older. Sixty-five percent of all dual eligibles had 2 or more chronic conditions, and among dual eligibles aged 65 or older with physical disabilities and cognitive limitations, 35% had 4 or more, with hypertension and arthritis the most common conditions. Dual eligibles aged 18 to 64 who had a usual source of medical care had a 127% increased likelihood of having MCC compared with those who did not have a usual source of care.

**Conclusion:**

Attention to disability can be a component to helping further understand the relationship between health and chronic conditions for dual eligible populations and other segments of our society with complex health and medical needs.

## Introduction

A segment of the population drawing scrutiny in recent years is the “dual eligibles,” the approximately 9 million low-income senior citizens and low-income people with disabilities as determined by the Social Security Administration (SSA) who are eligible for both Medicare and Medicaid coverage. Medicare enrollment provides primary insurance coverage for acute care medical services, and concurrent Medicaid enrollment helps them pay for premiums and services not fully covered by Medicare (1–13). Although the scope of their joint coverage varies according to their Medicaid eligibility, about 74% of dual eligibles under the age of 65 years receive full “wrap around” Medicaid coverage, as do 81% of dual eligibles aged 65 or older (6). The costs incurred to both Medicare and Medicaid by dual eligibles is disproportionate to their enrollment in these programs (1,7), leading to efforts to understand their use of health care to better inform policy and public health practice (8–13).

The health care associated with the high prevalence of chronic illness among dual eligibles accounts for much of these high costs (6,14). Seventy-two percent of dual eligibles have 2 or more chronic conditions, and 14% of dual eligibles with 6 or more chronic conditions account for 46% of Medicare spending (14). Having more than 1 chronic condition, however, frequently linked in some way to disability, does not mean that people cannot maintain their independence or lead quality lives (15–18). These findings have led to research examining the relationship between multiple chronic conditions (MCC) among dual eligibles and how these co-existing conditions are influenced by or contribute to functional disability limitations (19–21). The SSA definition of disability focuses on persons aged less than 65 with certain physical or mental conditions severe enough to create an inability to work (“substantial gainful activity”) in an ongoing capacity for at least a year or until death (22). Disability can be defined by using population-based data to describe functional limitations not directly related to work among this population also (19,20,23). Among working-age (18–64 years) adults with disabilities identified through self-reported limitations, more than half (an estimated 13 million adults) have more than 1 chronic condition. Compared with people without disabilities, when adjusted for MCC, people with disabilities have much higher health care use even as they experience greater difficulties in accessing care such as preventive services (20,24) or treatment that may be related to the conditions themselves (19,23). People with disabilities experience disparities in health status or receipt of preventive services that could be associated with 1 or more chronic conditions. These disparities may be related to work among younger cohorts of dual eligibles and to increased independence among older cohorts of dual eligibles (21). For some, these disparities exist because certain disabilities predispose them to chronic illnesses. For many, however, a likely explanation is that these disparities result from social, environmental, or behavioral determinants of health that can affect people with disabilities disproportionate to their distribution in the general population. In work commissioned by the US Department of Health and Human Services Strategic Initiative to identify the connection between functional disability limitations and MCC, 63% of dual eligibles living in community settings were found to have both chronic conditions and functional limitations, and dual eligibles with chronic conditions and functional limitations represented 1.5% of the US community-based population while accounting for 6% of national health care expenditures ($60.5 billion) (19).

The objective of our study was to build upon this earlier work by using a public health surveillance tool, the Medical Expenditure Panel Survey (MEPS), to better understand the relationship between functional disability limitations and MCC within a high-cost segment of the US publicly insured population, the dual eligibles. Our study is driven by 2 research questions: 1) what is the prevalence of MCC for discrete socioeconomic and health characteristics among dual eligibles in the United States overall and stratified by age category and type of disability limitation? and 2) what are the adjusted odds of having MCC among dual eligibles given sociodemographic and health characteristics, stratified by age category and type of disability limitation?

## Methods

The MEPS is an annual panel survey that collects demographic and health care expenditure and use information on individuals and families, including their health care providers, their employers, and their health care coverage (25). It is used to track health care services among subsets of the population, including people with disabilities (26–29). Multiple studies have demonstrated the use of the MEPS for analyzing health disparities among and between disability groups (16,18,24,26–30). Broad disability type is identified, allowing for grouping people with physical disabilities or cognitive limitations or both while accommodating for people who may not be able to respond themselves because of their cognitive limitation through proxy response. Accuracy of Medicare and Medicaid eligibility using the MEPS is optimized by first characterizing the difference between Medicare, Medicaid, and other public insurance for respondents and then showing respondents an actual Medicare or Medicaid card from that person’s state of residence.

To achieve sufficient sample size for our analyses, we pooled data for continuous years 2005 through 2010. Noninstitutionalized adults aged 18 years or older were identified as dual eligibles if they had “coverage at any time” for both Medicare and Medicaid during the year in which they were included (25). This resulted in an unweighted sample of 4,579 (weighted sample, 5,166,380).

Because of the unique characteristics associated with dual eligibles below age 65 and those 65 or older, we stratified our analyses by these age categories, further constructing 3 mutually exclusive groups for subsamples based on their having 1) physical disabilities, 2) physical disabilities and cognitive limitations, or 3) other disabilities. A physical disability was flagged if, at any time in the 2 years that a person was followed, the person reported walking limitations, using an assistive device, or needing assistance with activities of daily living (ADLs). People with cognitive limitations were those reporting confusion or memory loss, having problems making decisions, or requiring supervision for their own safety. Although too small a subgroup to study on its own because of insufficient cell sizes, we created a distinct subgroup of those with both physical and cognitive disabilities to account for people with multiple disabilities. Those not reporting a physical disability or having only a cognitive limitation or other disability comprised the final group, “other disabilities.” This group included people with disabilities such as mental health or cognitive limitations only. MCC were 2 or more chronic conditions of those included in the MEPS: heart attack, coronary heart disease, angina, angina pectoris, other heart disease (these 5 grouped as cardiovascular disease), stroke, emphysema, hypertension, diabetes, arthritis, and asthma. This is a subset of conditions used elsewhere (21,30). Overall, 65.8% of dual eligibles had MCC, an unweighted sample of 3,094 (weighted sample, 3,398,939).

Descriptive analyses determined prevalence of MCC and sociodemographic and health characteristics of the MCC and dual eligible population, identified by disability type. Logistic regression analyses weighted by population were used to assess odds ratios for having MCC within each age and disability category for all dual eligibles with MCC, adjusted for sociodemographic and health characteristics.

## Results

Fifty-three percent of dual eligibles aged 18 to 64 had MCC compared with 73.5% of those 65 or older (Table 1). Among these dual eligibles with MCC, mean ages and total annual expenditures were 51 and $18,137 for those aged 18 to 64 and 75 and $14,364 for those aged 65 or older, respectively. Among dual eligibles with MCC who had a physical disability alone, 68% had MCC among those aged 18 to 64 and 86% had MCC among those 65 or older, comparable to prevalence for those with both physical disabilities and cognitive limitations but higher than prevalence for these age groups for those not reporting a physical disability or having only a cognitive limitation or other disability (30% and 52%, respectively). The number of chronic conditions included in our definition of MCC ranged from 2 (24%) to 7 (<1%), with variation by disability and age categories (Figure). Sixty-six percent of all dual eligibles had 2 or more chronic conditions. Among dual eligibles aged 65 or older with physical disabilities and cognitive limitations, 35% had 4 or more chronic conditions. Dual eligibles aged 18 to 64 in each disability category had higher rates of 1 or fewer chronic conditions than did older dual eligibles.

**Table 1:** Prevalence of Multiple Chronic Conditions^a^ (MCC) Among of Medicare- and Medicaid-Eligible, Noninstitutionalized Adults in the United States, Overall and by Disability type, Medical Expenditure Panel Survey (MEPS), 2005–2010^b^

Characteristic	Physical Disability Only,^c^ N = 2,450,911		Physical Disability and Cognitive Limitation,^d^ N = 785,906		Other Disabilities,^e^ N = 1,929,562		Total, N = 5,166,380	
	18–64, N = 869,399	≥65, N = 1,581,512	18–64, N = 365,088	≥65, N = 420,818	18–64, N = 755,006	≥65, N = 1,174,556	18–64, N = 1,989,494	≥65, N = 3,176,886
**Multiple chronic conditions, % (95% CI)**	68.0 (62.4–73.1)	86.0 (83.4–88.2)	66.6 (58.2–74.1)	86.2 (80.7–90.3)	30.3 (25.2–35.8)	52.3 (47.9–56.6)	53.4 (49.5–57.2)	73.5 (70.8–76.1)
**Age, y, mean (95% CI)**	52.6 (51.6–53.6)	75.1 (74.7–75.6)	51.4 (49.8–53.0)	77.3 (76.2–78.4)	47.9 (46.1–49.8)	73.4 (72.9–73.9)	51.3 (50.5–52.1)	75.0 (74.6–75.4)
**Total annual health expenditures, mean (95% CI), $**	19,873 (15,229–24,518)	14,921 (13,646–16,196)	20,640 (16,376–24,905)	21,350 (17,450–25,250)	8,021 (6,126–9,916)	4,931 (4,351–5,512)	18,137 (16,227–20,047)	14,364 (13,187–15,541)

a Defined as 2 or more of the 7 identified by using MEPS in this analysis (heart attack, coronary heart disease, angina, angina pectoris, other heart disease [these 5 grouped as “cardiovascular”], stroke, emphysema, hypertension, diabetes, arthritis, and asthma). For chronic conditions, the full year consolidated files for MEPS include only questions related to these conditions.

b All point estimates are weighted.

c Includes those with functional limitations or those who use assistive devices. This means anyone who reported having long-term walking limitations, long-term need for assistive device, or long-term need for assistance with activities of daily living but did not report cognitive limitation.

d Includes those who reported having both a cognitive limitation and a physical disability. Cognitive limitations included those who reported confusion or memory loss, having problems making decisions, or requiring supervision for their own safety.

e Includes anyone who reported cognitive limitations alone or other types of disabilities such as those related to mental health.

**Figure:**
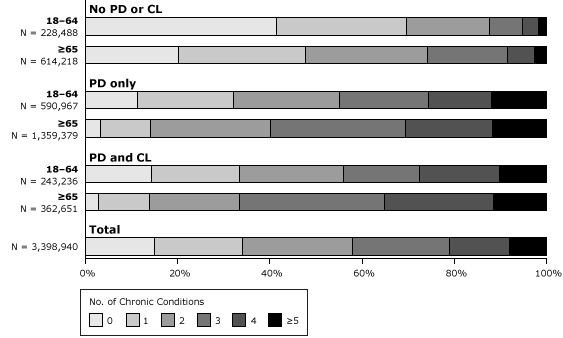
Multiple chronic conditions among Medicare- and Medicaid-eligible noninstitutionalized adults, by disability category and age, Medical Expenditure Panel Survey (MEPS), 2005–2010 (N = 3,398,940 [weighted]). Abbreviations: PD, physical disability; CL, cognitive limitation. The PD category includes those with functional limitations or those who use assistive devices. It includes anyone who reported having long-term walking limitations, long-term need for assistive device, or long-term need for assistance with activities of daily living but did not report cognitive limitation. CL includes those who reported confusion or memory loss, having problems making decisions, or requiring supervision for their own safety. The category “no PD or CL” includes those who did not identify as having either a PD or CL. For chronic conditions, the full-year consolidated files for MEPS include only questions related to heart attack, coronary heart disease, angina, angina pectoris, other heart disease (these 5 grouped as cardiovascular disease), stroke, emphysema, hypertension, diabetes, arthritis and asthma. **Table:** 

Category/Age	No. of Adults	No. of Chronic Conditions					
		0	1	2	3	4	≥5
**No PD or CL**							
18–64	228,488	41	28	18	7	4	2
≥65	614,218	20	28	26	17	6	3
**PD only**							
18–64	590,967	11	21	23	19	14	12
≥65	1,359,379	3	11	26	29	19	12
**PD and CL**							
18–64	243,236	14	19	23	17	17	10
≥65	362,651	3	11	20	31	24	11
**Total**	3,398,940	15	19	24	21	13	8

For dual eligibles aged 18 to 64 with MCC, 59% were female, 60% non-Hispanic white, and 10% Hispanic; 76% lived in an urban area, 50% lived below federal poverty guidelines, 31% lacked a high school diploma, and 29% reported poor health status (Table 2). Additionally, 59% were obese, 94% reported having a usual source of medical care, 74% were not married, and a greater percentage lived in the South (32%) than in other parts of the country. For dual eligibles with MCC who were aged 65 or older, 71% were female, 45% were non-Hispanic white, 22% were Hispanic, 81% lived in urban area, 35% lived below federal poverty guidelines, 61% lacked a high school diploma, and 17% reported poor health status. Thirty-nine percent were obese, with usual sources of care, married status, and residence in the South comparable to those aged less than 65 years.

**Table 2:** Sociodemographic and Health Characteristics of Medicare- and Medicaid-Eligible, Noninstitutionalized Adults with Multiple Chronic Conditions^a^ in the United States, by Disability and Age, Medical Expenditure Panel Survey (MEPS), 2005–2010^b^

Characteristic	Physical Disability Only,^c^ N = 1,950,346		Physical Disability and Cognitive Limitation,^d^ N = 605,887		Other Disabilities,^e^ N = 842,707		Total, N = 3,398,940	
	18–64, N = 590,967, % (95 CI)	≥65, N = 1,359,379, % (95 CI)	18–64, N = 243,236, % (95 CI)	≥65, N = 362,651, % (95 CI)	18–64, N = 228,488, % (95 CI)	≥65, N = 614,219, % (95 CI)	18–64, N = 1,062,692, % (95 CI)	≥65, N = 2,336,248, % (95 CI)
**Chronic condition**								
Arthritis	82.0 (77.6–85.7)	84.0 (81.1–86.5)	78.8 (72.6–83.9)	79.2 (73.2–84.1)	59.2 (49.6–68.2)	66.4 (61.5–70.9)	76.4 (73.0–79.5)	78.6 (76.0–81.0)
Asthma	35.2 (28.9–42.0)	21.8 (18.8–25.3)	44.6 (36.3–53.2)	18.8 (13.5–25.6)	29.6 (22.1–38.4)	14.6 (11.6–18.3)	36.1 (31.5–41.0)	19.5 (17.2–22.0)
Cardiovascular disease	42.2 (36.2–48.4)	52.8 (49.0–56.6)	40.7 (33.4–48.3)	63.5 (57.0–69.5)	37.1 (29.6–45.2)	41.1 (36.0–46.4)	40.8 (36.5–45.2)	51.4 (48.3–54.5)
Diabetes	45.3 (39.5–51.1)	41.6 (37.8–45.6)	44.9 (36.2–54.0)	35.8 (29.0–43.3)	39.1 (31.1–47.7)	38.4 (33.1–43.9)	43.9 (39.5–48.3)	39.9 (36.8–43.0)
Emphysema	17.2 (12.7–23.0)	12.5 (10.0–15.4)	11.1 (7.4–16.4)	14.5 (9.1–22.3)	9.7 (6.0–15.4)	11.3 (7.9–15.9)	14.2 (11.3–17.8)	12.5 (10.5–14.8)
Hypertension	84.8 (80.0–88.7)	89.8 (87.4–91.7)	77.4 (70.2–83.2)	89.9 (82.7–91.6)	79.7 (71.7–85.9)	88.7 (85.0–91.7)	82.0 (77.9–85.5)	89.2 (87.3–90.8)
Stroke	18.8 (14.1–24.6)	20.5 (17.8–23.5)	29.3 (22.6–37.2)	36.2 (29.4–43.7)	11.9 (8.3–16.9)	12.0 (8.2–17.1)	19.7 (16.3–23.6)	20.7 (18.5–23.2)
**Have a usual source of medical care**								
No	5.9 (3.7–9.2)	4.8 (3.5–6.4)	2.4 (1.5–4.0)	5.7 (2.9–11.1)	10.7 (5.9–18.6)	7.3 (5.0–10.7)	6.1 (4.3–8.6)	5.6 (4.4–7.0)
Yes	94.1 (90.8–96.3)	95.2 (93.6–96.5)	97.6 (96.0–98.5)	94.3 (88.9–97.1)	89.3 (81.4–94.1)	92.7 (89.3–95.0)	93.9 (91.4–95.7)	94.4 (93.0–95.6)
**Age, y**								
18–34	4.5 (2.3–8.5)	NA	3.1 (1.0–9.3)	NA	12.8 (7.5–20.9)	NA	6.0 (4.0–8.8)	NA
35–54	47.5 (41.4–53.7)		55.1 (47.2–62.7)		60.8 (52.5–68.5)		52.1 (47.8–56.4)	
55–64	48.0 (42.2–53.8)		41.8 (34.2–49.8)		26.4 (19.8–34.3)		41.9 (37.7–46.3)	
65–84	NA	81.3 (79.1–84.1)	NA	72.3 (63.5–79.6)	NA	94.2 (92.1–95.8)	NA	83.3 (80.7–85.5)
≥85		18.7 (15.9–21.9)		27.7 (20.4–36.5)		5.8 (4.2–7.9)		16.7 (14.5–19.3)
**Race/ethnicity**								
Non-Hispanic white	61.7 (55.3–67.8)	46.3 (41.9–50.9)	56.0 (47.7–64.0)	43.9 (36.2–51.9)	57.4 (48.6–65.8)	41.0 (35.4–46.7)	59.5 (54.6–64.3)	44.6 (40.7–48.5)
Non-Hispanic black	21.8 (17.7–26.5)	24.2 (21.3–27.2)	27.6 (21.0–35.3)	21.6 (16.6–27.7)	27.6 (21.1–35.3)	17.0 (13.7–20.8)	24.4 (20.8–28.4)	21.9 (19.5–24.5)
Other non-Hispanic	6.1 (3.0–12.0)	9.8 (7.4–12.5)	5.0 (2.5–9.9)	12.8 (8.6–18.6)	5.9 (2.5–13.4)	14.0 (10.2–18.9)	5.8 (3.5–9.4)	11.3 (9.1–13.8)
Hispanic	10.3 (7.3–14.4)	19.9 (16.4–23.8)	11.4 (7.8–16.2)	21.7 (16.0–28.8)	9.1 (5.4–14.9)	28.1 (23.2–33.5)	10.3 (8.0–13.1)	22.3 (19.1–25.8)
**Sex**								
Male	38.4 (32.6–44.6)	27.7 (24.3–31.3)	36.3 (28.8–44.6)	18.9 (13.3–26.3)	53.5 (45.2–61.7)	38.1 (32.8–43.8)	41.2 (36.6–45.8)	29.1 (26.3–32.0)
Female	61.6 (55.4–67.4)	72.3 (68.7–75.7)	63.7 (55.4–71.2)	81.1 (73.7–86.7)	46.5 (38.3–54.8)	61.9 (56.2–67.2)	58.8 (54.2–63.4)	70.9 (68.0–73.7)
**Education**								
Less than high school diploma	30.3 (25.3–35.7)	60.2 (56.1–64.2)	31.8 (24.6–40.0)	62.1 (53.4–70.2)	33.9 (26.3–42.5)	62.0 (56.5–67.2)	31.4 (27.6–35.5)	61.0 (57.6–64.3)
High school diploma or equivalent	56.1 (50.6–61.5)	31.1 (27.7–34.7)	54.2 (46.3–61.9)	26.4 (19.7–34.3)	59.7 (50.9–67.9)	26.4 (21.4–32.2)	56.4 (52.2–60.6)	29.1 (26.2–32.2)
More than high school diploma	13.6 (10.4–17.6)	8.7 (6.2–12.1)	14.0 (9.5–20.2)	11.5 (6.4–19.8)	6.4 (2.7–14.4)	11.6 (8.3–15.8)	12.2 (9.6–15.3)	9.9 (7.4–13.0)
**Married**								
No	73.0 (67.8–77.6)	73.7 (68.9–78.0)	77.6 (68.5–84.6)	76.4 (68.7–82.7)	71.8 (62.4–79.6)	62.3 (56.5–67.8)	73.8 (69.7–77.5)	71.1 (67.1–74.9)
Yes	27.0 (22.4–32.2)	26.3 (22.0–31.1)	22.4 (15.4–31.5)	23.6 (17.3–31.3)	28.2 (20.4–37.6)	37.7 (32.2–43.5)	26.2 (22.5–30.3)	28.9 (25.1–32.9)
**Income^f^**								
Poor	50.2 (44.7–55.8)	37.8 (34.2–41.6)	57.8 (49.0–66.1)	29.4 (23.6–35.9)	39.5 (32.1–47.5)	31.1 (26.5–36.1)	49.7 (45.3–54.1)	34.7 (31.7–37.9)
Not Poor	49.8 (44.2–55.3)	62.2 (58.4–65.8)	42.2 (33.9–51.0)	70.6 (64.1–76.4)	60.4 (52.5–67.9)	68.9 (63.9–73.5)	50.3 (45.9–54.7)	65.3 (62.1–68.3)
**Body mass index^g^**								
Underweight	2.1 (1.3–3.3)	3.4 (2.4–4.9)	0	3.1 (1.5–6.3)	1.1 (0.3–3.4)	1.9 (0.8–4.2)	1.4 (0.9–2.1)	3.0 (2.2–4.0)
Normal weight	17.2 (12.2–23.6)	24.0 (21.0–27.2)	23.4 (17.0–31.4)	34.9 (29.0–41.3)	15.8 (10.5–23.3)	29.7 (25.1–34.7)	18.3 (14.7–22.6)	27.2 (24.9–29.6)
Overweight	21.2 (17.0–26.1)	27.8 (25.0–30.9)	17.3 (12.2–23.9)	28.2 (22.1–35.2)	25.7 (18.8–34.1)	40.9 (35.8–46.1)	21.3 (17.7–25.3)	31.3 (29.1–33.6)
Obese	59.5 (53.6–65.2)	44.8 (41.0–48.7)	59.3 (51.3–66.8)	33.8 (27.7–40.4)	57.4 (48.2–66.1)	27.6 (23.0–32.6)	59.0 (54.4–63.5)	38.5 (35.7–41.4)
**Health status**								
Excellent	1.8 (0.8–4.3)	3.9 (2.3–6.4)	0.2 (0.0–1.5)	1.7 (0.8–3.6)	4.3 (1.7–10.4)	7.4 (5.1–10.7)	2.0 (1.1–3.6)	4.5 (3.2–6.1)
Very good	6.1 (4.0–9.1)	12.1 (9.8–14.8)	4.2 (2.3–7.3)	8.0 (5.0–12.7)	7.4 (4.3–12.3)	23.7 (19.7–28.2)	5.9 (4.4–7.9)	14.5 (12.6–16.7)
Good	22.4 (18.3–27.0)	29.7 (26.6–32.9)	17.5 (12.0–24.9)	19.7 (15.3–24.9)	42.4 (34.9–50.2)	39.3 (34.6–44.3)	25.6 (22.4–29.0)	30.7 (28.3–33.1)
Fair	37.7 (33.6–42.0)	36.5 (33.5–39.6)	39.3 (32.7–46.3)	35.8 (29.2–43.0)	35.1 (28.4–42.5)	25.6 (21.8–29.8)	37.5 (34.7–40.4)	33.5 (31.3–35.8)
Poor	32.0 (27.2–37.2)	17.9 (15.1–21.0)	38.8 (31.6–46.6)	34.8 (28.2–42.0)	10.9 (6.6–17.4)	4.0 (2.4–6.6)	29.0 (25.3–33.0)	16.8 (14.8–19.1)
**Urban residence^h^**								
Non-MSA	24.6 (18.9–31.3)	19.9 (16.5–23.8)	23.9 (16.9–32.6)	21.3 (15.3–28.8)	20.1 (14.5–27.2)	16.3 (12.1–21.5)	23.5 (18.9–28.7)	19.2 (16.0–22.8)
MSA	75.4 (68.7–81.1)	80.1 (76.2–83.5)	76.1 (67.4–83.1)	78.7 (71.2–84.7)	79.9 (72.8–85.5)	83.7 (78.5–87.9)	76.5 (71.3–81.1)	80.8 (77.2–84.0)
**US region**								
Northeast	18.0 (13.3–23.8)	21.0 (16.8–25.8)	18.1 (12.0–26.5)	18.9 (12.7–27.1)	11.9 (7.2–19.1)	25.5 (20.6–31.1)	16.7 (13.1–21.0)	21.8 (18.5–25.5)
Midwest	31.9 (26.0–38.5)	19.8 (16.5–23.6)	21.8 (15.8–29.4)	10.3 (6.8–15.3)	28.7 (21.1–37.7)	14.9 (11.0–19.8)	28.9 (24.8–33.4)	17.0 (14.6–19.7)
South	31.5 (26.1–37.4)	36.6 (32.1–41.4)	22.7 (17.0–29.6)	38.2 (30.5–46.6)	41.8 (32.7–51.4)	32.5 (27.4–38.1)	31.7 (27.7–36.0)	35.8 (31.9–39.9)
West	18.6 (13.3–25.5)	22.6 (18.8–26.9)	37.3 (30.0–45.4)	32.6 (25.5–40.6)	17.6 (11.0–26.8)	27.2 (22.0–33.0)	22.7 (18.7–27.2)	25.3 (21.6–29.5)

Abbreviation: NA, not applicable.

a Defined as 2 or more of the 7 identified by using MEPS in this analysis (heart attack, coronary heart disease, angina, angina pectoris, other heart disease [these 5 grouped as “cardiovascular”], stroke, emphysema, hypertension, diabetes, arthritis, and asthma). For chronic conditions, the full year consolidated files for MEPS include only questions related to these conditions.

b All point estimates are weighted.

c Includes those with functional limitations or those who use assistive devices. This means anyone who reported having long-term walking limitations, long-term need for assistive device, or long-term need for assistance with activities of daily living but did not report cognitive limitation.

d Includes those who reported having both a cognitive limitation and a physical disability. Cognitive limitations included those who reported confusion or memory loss, having problems making decisions, or requiring supervision for their own safety.

e Includes anyone who reported cognitive limitations alone or other types of disabilities such as those that were related to mental health.

f Income is computed (by the Agency for Healthcare Research and Quality) as family income as a percentage of the poverty line (http://meps.ahrq.gov/survey_comp/hc_technical_notes.shtml, accessed August 5, 2013). People were considered to have “poor” income if they reported an income that fell into the category of “poor/negative.”

g Body mass index (BMI) categories from the National Heart, Lung, and Blood Institute (http://www.nhlbi.nih.gov/health/public/heart/obesity/lose_wt/risk.htm, accessed December 26, 2012). These are calculated using the following formula: [weight in pounds ÷ (height in inches × height in inches)] × 703. Underweight was defined as BMI of less than 18.5; normal weight was BMI of 18.5 or higher and less than 25; overweight was BMI of 25 or higher and less than 30; obese was BMI of 30 or higher.

h Metropolitan statistical areas (MSAs) have at least 1 urbanized area of 50,000 or more population plus adjacent territory that has a high degree of social and economic integration with the core as measured by commuting ties (http://www.whitehouse.gov/sites/default/files/omb/assets/omb/bulletins/fy2009/09-01.pdf, accessed December 26, 2012).

The leading chronic conditions among those measured for both those aged 18 to 64 and those 65 or older were hypertension and arthritis, respectively. Among each disability category and the total, the greatest differences between those aged 18 to 64 years and those aged 65 or older were for having asthma and cardiovascular disease, with 36% of all those aged 18 to 64 reporting asthma compared with 20% of those 65 or older and 41% of those 18 to 64 reporting cardiovascular conditions compared with 51% of those 65 or older.

Variation in prevalence was evident between subsets of this population. Hispanics comprised a larger proportion of dual eligibles with MCC among those aged 65 or older compared with those aged 18 to 64, regardless of disability category. Alternately, non-Hispanic whites comprised a substantially larger proportion of those aged 18 to 64 than of those aged 65 or older. The proportion of women was higher than the proportion of men among those with MCC, regardless of age, but this differential was larger for those aged 65 or older. In education, twice the proportion of dual eligibles with MCC aged 65 or older had less than a high school education than seen among those aged 18 to 64. For dual eligibles with a high school education, there were higher proportions in the younger group than in the older group. Dual eligibles aged 18 to 64 in each disability category had high obesity rates, as high as 60% among those with physical disabilities. High percentages of poor health status were reported by those aged 18 to 64, with the highest rate (39%) among dual eligibles with both physical disabilities and cognitive limitations; among those aged 65 or older, poor health status was highest among those with both physical disabilities and cognitive limitations. A greater proportion of dual eligibles with MCC aged 65 or older lived in the South than in other regions of the country.

Several factors were significantly associated with MCC. Among all dual eligibles aged 18 to 64 having MCC, men aged 18 to 64 were 25% less likely to have MCC than women (Table 3). With each year of age within this age category, the odds of MCC increased by 9%, and Hispanics were significantly less likely than non-Hispanic whites to have MCC. In this age group for all dual eligibles, there was a 59% or 69% greater likelihood of having MCC among those living in the Midwest or South, respectively, compared with those in the West, a 31% greater likelihood among nonurban dwellers, and a 127% increased odds among those with a usual source of medical care. Fair or poor health status and obesity or underweight were associated with MCC in this age category, as they were for those aged 65 or older. Significant odds ratios for dual eligibles among those aged 65 or older suggested a greater likelihood of MCC for age, being non-Hispanic black, having a high school or lower education, having a usual source of medical care, obesity or underweight, and fair or poor health status.

**Table 3:** Adjusted Odds Ratios of Having Multiple Chronic Conditions^a^ Among Medicare- and Medicaid-Eligible Noninstitutionalized Adults, by Disability and Age Categories, Medical Expenditure Panel Survey (MEPS), 2005–2010^b^

Characteristic	Physical Disability Only,^c^ N = 1,950,346		Physical Disability and Cognitive Limitation,^d^ N = 605,887		Other Disabilities^e^, N = 842,707		Total, N = 3,398,940	
	18–64, N = 590,967, OR (95% CI)	≥65, N = 1,359,379, OR (95% CI)	18–64, N = 243,236, OR (95% CI)	≥65, N = 362,651, OR (95% CI)	18–64, N = 228,488, OR (95% CI)	≥65, N = 614,219, OR (95% CI)	18–64, N = 1,062,692, OR (95% CI)	≥65, N = 2,336,248, OR (95% CI)
**Sex**								
Male	0.74 (0.46–1.18)	1.41 (0.84–2.35)	1.11 (0.49–2.54)	0.33 (0.12–0.90)	1.30 (0.74–2.28)	0.89 (0.62–1.28)	0.76 (0.64–0.88)	0.94 (0.86–1.03)
Female	1 [Reference]							
**Age,^f^ mean**	1.05 (1.02–1.08)	1.00 (0.97–1.03)	1.13 (1.08–1.19)	0.99 (0.94–1.05)	1.08 (1.05–1.10)	1.00 (0.97–1.04)	1.09 (1.09–1.10)	1.05 (1.04–1.06)
**Race/ethnicity**								
Non-Hispanic white	1 [Reference]							
Non-Hispanic black	1.08 (0.64–1.82)	1.21 (0.64–2.26)	3.89 (1.47–10.33)	0.75 (0.22–2.52)	1.53 (0.84–2.79)	0.77 (0.47–1.26)	0.91 (0.75–1.10)	1.23 (1.05–1.43)
Other non-Hispanic	1.28 (0.49–3.35)	1.09 (0.50–2.41)	3.76 (0.80–17.68)	0.26 (0.07–0.97)	0.85 (0.29–2.48)	0.67 (0.35–1.31)	1.10 (0.80–1.51)	0.87 (0.68–1.12)
Hispanic	0.86 (0.36–2.05)	0.44 (0.24–0.80)	2.21 (0.75–6.53)	0.54 (0.18–1.66)	0.80 (0.40–1.62)	0.67 (0.39–1.13)	0.65 (0.52–0.82)	0.72 (0.60–0.86)
**Education**								
Less than high school diploma	1.24 (0.60–2.55)	1.60 (0.69–3.71)	2.31 (0.57–9.40)	0.72 (0.14–3.64)	1.02 (0.30–3.45)	1.45 (0.81–2.58)	1.12 (0.89–1.46)	1.35 (1.17–1.55)
High school diploma or equivalent	1.12 (0.54–2.30)	1.20 (0.49–2.98)	4.60 (1.11–19.05)	2.87 (0.40–20.71)	1.09 (0.32–3.71)	0.89 (0.46–1.72)	1.15 (0.91–1.46)	1.15 (1.03–1.30)
More than high school diploma	1 [Reference]							
**Income^g^**								
Poor	1.19 (0.72–1.95	1.37 (0.86–2.16)	1.82 (0.87–3.79)	0.78 (0.40–1.53)	0.69 (0.44–1.09)	1.15 (0.83–1.60)	0.96 (0.83–1.10)	0.98 (0.85–1.12)
Not Poor	1 [Reference]							
**Married**								
Yes	1 [Reference]							
No	0.55 (0.33–0.93)	1.55 (0.94–2.55)	0.27 (0.09–0.83)	0.61 (0.20–1.86)	0.93 (0.44–2.00)	0.92 (0.62–1.38)	0.97 (0.83–1.14)	1.06 (0.95–1.18)
**US region**								
Northeast	1.55 (0.62–3.85)	1.54 (0.73–3.22)	1.37 (0.38–4.90)	1.18 (0.36–3.88)	0.45 (0.18–1.12)	1.50 (0.89–2.54)	1.13 (0.90–1.43)	0.99 (0.84–1.18)
Midwest	2.30 (1.09–4.86)	1.30 (0.57–2.98)	1.00 (0.29–3.44)	3.61 (0.70–18.58)	1.41 (0.52–3.80)	2.46 (1.26–4.79)	1.59 (1.26–2.00)	0.99 (0.83–1.17)
South	1.02 (0.49–2.12)	1.15 (0.61–2.18)	0.17 (0.06–0.55)	2.40 (0.96–5.98)	1.14 (0.47–2.78)	1.54 (0.95–2.50)	1.69 (1.35–2.12)	1.09 (0.95–1.24)
West	1 [Reference]							
**Urban residence^h^**								
MSA	1 [Reference]							
Non-MSA	0.97 (0.52–1.80)	0.83 (0.48–1.41)	1.83 (0.70–4.80)	0.81 (0.30– 2.18)	0.58 (0.31–1.11)	0.90 (0.52–1.57)	1.31 (1.08–1.61)	0.94 (0.82–1.08)
**Health status**								
Excellent/very good/good	0.37 (0.24–0.50)	0.69 (0.46–1.03)	0.24 (0.10–0.58)	0.37 (0.16–0.85)	0.37 (0.20–0.67)	0.57 (0.39–0.85)	0.27 (0.24–0.31)	0.27 (0.24–0.31)
Fair/poor	1 [Reference]							
**Have a usual source of medical care**								
Yes	1.36 (0.67–2.74)	1.34 (0.61–2.94)	3.53 (0.90–13.82)	2.60 (0.67–10.08)	1.05 (0.44–2.53)	2.80 (1.64–4.79)	2.27 (1.88–2.75)	3.02 (2.52–3.63)
No	1 [Reference]							
**Healthy weight^i^**								
Yes	0.42 (0.27–0.65)	0.41 (0.25–0.68)	0.25 (0.12–0.51)	0.24 (0.11–0.53)	0.40 (0.22–0.73)	0.54 (0.34–0.87)	0.39 (0.34–0.46)	0.34 (0.31–0.39)
No	1 [Reference]							

Abbreviations: OR, odds ratio; CI, confidence interval.

a Defined as 2 or more of the 7 identified by using MEPS in this analysis (heart attack, coronary heart disease, angina, angina pectoris, other heart disease [these 5 grouped as “cardiovascular”], stroke, emphysema, hypertension, diabetes, arthritis, and asthma). For chronic conditions, the full year consolidated files for MEPS include only questions related to these conditions.

b All point estimates are weighted. The multiple logistic regressions using multiple chronic conditions as the dependent variable were controlled for individual’s sex, age, race, ethnicity, education, marital status, region of residence, rural or urban residence, health status, usual source of care status, and healthy weight, and included the intercept term. The likelihood ratio test comparing the predicted model to the null model indicated a significant improvement of fit.

c Includes those with functional limitations or those who use assistive devices. This means anyone who reported having long-term walking limitations, long-term need for assistive device, or long-term need for assistance with activities of daily living but did not report cognitive limitation.

d Includes those who reported having both a cognitive limitation and a physical disability. Cognitive limitations included those who reported confusion or memory loss, having problems making decisions, or requiring supervision for their own safety.

e Includes anyone who reported cognitive limitations alone or other types of disabilities such as those that were related to mental health.

f Change in OR for each year of age in the category.

g Income is computed (by the Agency for Healthcare Research and Quality) as family income as a percentage of the poverty line (http://meps.ahrq.gov/survey_comp/hc_technical_notes.shtml, accessed August 5, 2013). People were considered to have “poor” income if they reported an income that fell into the category of “poor/negative.”

h Metropolitan statistical areas (MSAs) have at least 1 urbanized area of 50,000 or more population plus adjacent territory that has a high degree of social and economic integration with the core as measured by commuting ties (http://www.whitehouse.gov/sites/default/files/omb/assets/omb/bulletins/fy2009/09-01.pdf, accessed December 26, 2012).

i Body mass index (BMI) categories from the National Heart, Lung, and Blood Institute (http://www.nhlbi.nih.gov/health/public/heart/obesity/lose_wt/risk.htm, accessed December 26, 2012). These are calculated using the following formula: [weight in pounds ÷ (height in inches × height in inches)] × 703. Healthy weight is defined as having a body mass index of greater than 18.5 and less than 30. Underweight (<18.5) was under 4% in all of the categories studied.

Among dual eligibles with only physical disabilities, significant odds ratios associated with having MCC were found among those aged 18 to 64 for age, being married, living in the Midwest compared with the West, reporting fair or poor health, or not having a healthy weight. Except for health status and healthy weight, all of these significant associations disappeared for dual eligibles with physical disabilities who were 65 or older, with only non-Hispanic ethnicity being significant. Among people with both physical and cognitive disabilities, significant odds of having MCC were found, again among those aged 18 to 64 years first, for age, being black, being a high school graduate, being married, living in the South, reporting fair or poor health status, or unhealthy weight. For those 65 or older with both physical and cognitive limitations, significant odds were found for women, other race/ethnicity (this includes Asian, American Indian, and persons from the Pacific Rim), reporting fair or poor health status, or unhealthy weight. For all other dual eligibles (“other disabilities” category), age, fair or poor health status, and unhealthy weight were significant among those aged 18 to 64 years, with Midwest residence replacing age as significant also among those 65 or older.

## Discussion

Most attention to dual eligibles has been tied in some way to controlling their cost to the health care system, leading to discussions of targeted care management strategies, early screening for potential high cost or high risk conditions to apply preventive strategies, and any number of risk-adjustment schema that help provide more equitable compensation for high-end and high-resource services. Attention has also focused on better understanding the scope and range of chronic conditions among dual eligibles, with the implications that greater attention to controlling 1 or more of the conditions through a multidisciplinary delivery model may prove beneficial. Our work amplifies a third factor that can be considered: disability, or more broadly, functionality as reflected by disability. Our findings show that sociodemographic or health-related characteristics may influence the likelihood of an association with MCC among all dual eligibles. One implication of this finding is the potential to more fully address the social determinants of health affecting those with certain types of physical disabilities in ways that may help control the effects of MCC through improved access to services, not just in health care settings but in communities at large, realizing the importance of targeting some of these strategies to work-related environments, especially for those under age 65. Regardless of whether the disability preceded or followed the chronic conditions identified in this research, greater environmental accessibility to physical activities, adequate nutrition, health care services, disease management education, and other components that directly address the ability of dual eligibles to function more freely within communities could improve outcomes. Given our findings that suggest high rates of usual sources of medical care for all disability categories among dual eligibles, provider education may also play a role.

MEPS data are limited in ways that influence the ability to extrapolate findings. Responses are self-reported, which can result in errors related to recall or poor understanding. Lack of understanding is made more likely by the representation of people with functional disabilities in the dual eligible population, many of whom have cognitive disabilities that may influence responses. Although MEPS routinely uses proxies as respondents for those with cognitive limitations, this methodology can increase bias because the person reporting may not have accurate knowledge of experiences solicited by the questions. In addition, the questions used to identify cognitive limitations are broad, including a wide range of disability from intellectual disability to dementia. Because MEPS does not oversample on the basis of disability, some less common disabilities may not be fully represented within the dual eligible population.

Our sample only includes dual eligibles from the noninstitutionalized population, while many, estimated at 17% (7), of dual eligibles live in institutional settings. This limitation makes comparisons to studies using other data sources that include people who live in large congregate care facilities somewhat problematic, and we assume that for this reason some of our prevalence rates may be underestimated. In addition, our selection of the 7 most frequently identified chronic conditions is a subset of others identified elsewhere (14,17,18). Although we thought the 7 conditions represented a meaningful subset of chronic conditions pervasive in this population, direct comparisons with other research that included conditions such as, for example, depression, identified through data sources such as the Medicare Current Beneficiary Data, are not possible.

Limitations notwithstanding, our analysis shows that within the dual eligible population, disability subgroups have unique associations with MCC. People with physical disabilities, especially if accompanied by a cognitive limitation, are among the highest costing and sickest of our noninstitutionalized dual eligible population. The potential for more targeted interventions aligning with the goals of the US Department of Health and Human Services Strategic Initiative is promising. Such interventions would target improvement of overall health status by taking into account ongoing efforts to understand and address the challenges faced by the dual eligible population in accessing treatment of chronic conditions and heightened attention to identifying and targeting disability subgroups for whom strategies can be identified. A better understanding of dual eligibles that includes disability can help policy makers more effectively design cost-containment strategies, case managers more effectively steer consumer-directed health plans, and people enrolled in both Medicaid and Medicare identify practices among targeted and coordinated services that will lead to their improved health at substantially reduced costs to themselves and the emerging systems in which they will be a part.

## References

[1] Medicare Payment Advisory Commission. Health care spending and the Medicare program. Washington (DC); 2012. http://www.medpac.gov/documents/Jun12DataBookEntireReport.pdf. Accessed October 1, 2012.

[2] Yip J , Nishita CM , Crimmins EM , Wilber KH . High-cost users among dual eligibles in three care settings. J Health Care Poor Underserved 2007;18(4):950–65. 10.1353/hpu.2007.0109 17982217

[3] Verdier JM , Au M , Gillooly J . Managing the care of dual eligible beneficiaries: a review of selected state programs and special needs plans. Washington (DC): Mathematica Policy Research Inc; 2011. http://www.medpac.gov/documents/Jun11_ManagingDualEligibles_CONTRACTOR.pdf. Accessed November 12, 2012.

[4] Rousseau D , Clemans-Cope L , Lawton E , Langston J , Connolly J , Howard J . Dual eligibles: Medicaid enrollment and spending for Medicare beneficiaries in 2007. Washington (DC): Kaiser Commission on Medicaid and the Uninsured; 2010.

[5] Kaiser Commission on Medicaid Facts. Dual eligibles: Medicaid’s role for low-income Medicare beneficiaries. The Henry J. Kaiser Family Foundation; 2011. http://www.kff.org/medicaid/upload/4091-08.pdf. Accessed March 14, 2013.

[6] Kaiser Family Foundation. Medicare Policy Issue Brief. Medicare’s role for dual eligible beneficiaries; 2012. http://www.kff.org/medicare/upload/8138-02.pdf. Accessed March 14, 2013.

[7] Kaiser Commission on Medicaid and the Uninsured. The diversity of dual eligible beneficiaries: an examination of services and spending for people eligible for both Medicaid and Medicare. The Henry J. Kaiser Family Foundation; 2012. http://www.kff.org/medicaid/upload/7895-02.pdf. Accessed March 14, 2013.

[8] Clemans-Cope L , Waidmann T . Improving care for dual eligibles through innovations in financing. N Engl J Med 2011;365(11):e21. 10.1056/NEJMp1108571 21879892

[9] Grabowski DC . Care coordination for dually eligible Medicare-Medicaid beneficiaries under the Affordable Care Act. J Aging Soc Policy 2012;24(2):221–32. 10.1080/08959420.2012.659113 22497360

[10] Centers for Medicare and Medicaid Services. State demonstrations to integrate care for dual eligible individuals — design contracts. http://www.cms.gov/Medicare-Medicaid-Coordination/Medicare-and-Medicaid-Coordination/Medicare-Medicaid-Coordination-Office/StateDemonstrationstoIntegrateCareforDualEligibleIndividuals.html. Accessed April 9, 2013.

[11] Gold MR , Jacobson GA , Garfield RL . There is little experience and limited data to support policy making on integrated care for dual eligibles. Health Aff (Millwood) 2012;31(6):1176–85. 10.1377/hlthaff.2012.0162 22665829

[12] Neuman P , Lyons B , Rentas J , Rowland D . Dx for a careful approach to moving dual-eligible beneficiaries into managed care plans. Health Aff (Millwood) 2012;31(6):1186–94. 10.1377/hlthaff.2012.0160 22665830

[13] Letter to state Medicaid directors Re: Financial models to support state efforts to integrate care for Medicare-Medicaid enrollees. Baltimore (MD): Centers for Medicare and Medicaid Services; 2011. https://www.cms.gov/smdl/downloads/Financial_Models_Supporting_Integrated_Care_SMD.pdf. Accessed February 21, 2012.

[14] Centers for Medicare and Medicaid Services. Chronic conditions among Medicare beneficiaries, chartbook. Baltimore (MD); 2012. http://www.cms.gov/Research-Statistics-Data-and-Systems/Statistics-Trends-and-Reports/Chronic-Conditions/2012ChartBook.html. Accessed March 14, 2013.

[15] US Department of Health and Human Services. The Surgeon General’s call to action to improve the health and wellness of persons with disabilities. Rockville (MD): Public Health Service, Office of the Surgeon General; 2005. http://www.surgeongeneral.gov/library/calls/disabilities/index.html. Accessed August 5, 2013. 20669510

[16] Institute of Medicine. Living well with chronic illness: a call for public health action. Washington (DC): National Academies Press; 2012. http://books.nap.edu/openbook.php?record_id=13272. Accessed March 14, 2013.

[17] Parekh AK , Goodman RA , Gordon C , Koh HK ; HHS Interagency Workgroup on Multiple Chronic Conditions. Managing multiple chronic conditions: a strategic framework for improving health outcomes and quality of life. Public Health Rep 2011;126(4):460–71. 21800741 10.1177/003335491112600403 PMC3115206

[18] Goodman RA , Parekh AK , Koh HK . Toward a more cogent approach to the challenges of multimorbidity. Ann Fam Med 2012;10(2):100–1. 10.1370/afm.1391 22412000 PMC3315138

[19] Alecxih L , Shen S , Chan I , Taylor D , Drabek J . Individuals living in the community with chronic conditions and functional limitations: a closer look. Office of the Assistant Secretary for Planning and Evaluation, US Department of Health and Human Services; 2010. http://aspe.hhs.gov/daltcp/reports/2010/closerlook.htm. Accessed December 26, 2012.

[20] Reichard A , Fox MH . Using population-based data to examine preventive services by disability type among dually eligible (Medicare/Medicaid) adults. Disabil Health J 2013;6(2):75–86. 10.1016/j.dhjo.2012.12.001 23507157 PMC4478586

[21] Hall JP . Dually-eligible working-age adults with disabilities: issues and challenges as health reform is implemented. Disabil Health J 2013;6(2):72–4. 10.1016/j.dhjo.2013.01.004 23507156

[22] US Social Security Administration. 2013 red book: a summary guide to employment supports for persons with disabilities under the Social Security Disability Insurance and Supplemental Security Income programs. http://www.ssa.gov/redbook/eng/TheRedBook2013.pdf. Accessed March 7, 2013.

[23] Gulley SP , Rasch EK , Chan L . The complex web of health: relationships among chronic conditions, disability, and health services. Public Health Rep 2011;126(4):495–507. 21800744 10.1177/003335491112600406 PMC3115209

[24] Reichard A , Stolzle H , Fox MH . Health disparities among adults with physical disabilities or cognitive limitations compared to individuals with no disabilities in the United States. Disabil Health J 2011;4(2):59–67. 10.1016/j.dhjo.2010.05.003 21419369

[25] Agency for Healthcare Research and Quality. Medical Expenditure Panel Survey; 2013. http://meps.ahrq.gov/mepsweb/. Accessed April 2, 2013. 38416859

[26] National healthcare disparities report. Rockville (MD): Agency for Healthcare Research and Quality; 2009.

[27] Schootman M , Jeffe DB . Identifying factors associated with disability-related differences in breast cancer screening (United States). Cancer Causes Control 2003;14(2):97–107. 10.1023/A:1023091308625 12749715

[28] Reichard A , Stolzle H . Diabetes among adults with cognitive limitations compared to individuals with no cognitive disabilities. Intellect Dev Disabil 2011;49(3):141–54. 10.1352/1934-9556-49.2.141 21639741

[29] Rasch EK , Gulley SP , Chan L . Use of emergency departments among working age adults with disabilities: a problem of access and service needs. Health Serv Res 2013;48(4):1334–58. 10.1111/1475-6773.12025 23278461 PMC3724353

[30] Smith DL . Disparities in patient-physician communication for persons with a disability from the 2006 Medical Expenditure Panel Survey (MEPS). Disabil Health J 2009;2(4):206–15. Erratum in Disabil Health J 2010;3(2):130. 10.1016/j.dhjo.2009.06.002 21122761

