# Erratum, Vol. 10, September 19, 2013, Release

**DOI:** 10.5888/pcd11.130064e

**Published:** 2014-07-24

A correction has been made to a paragraph of the article “Disability, Health, and Multiple Chronic Conditions Among People Eligible for Both Medicare and Medicaid, 2005–2010.” The prevalence rates presented in Table 2 were inaccurately interpreted in the article. The authors inadvertently confused 1) the proportion of persons in each cell defined by age and another characteristic, which comprised persons with disabilities who had multiple chronic conditions (MCC) in the broader category, with 2) the proportion of persons in each category defined by age and another characteristic (in the column or row) who have MCC. This led to inappropriate comparisons between cell prevalence rates.

The fourth paragraph of the Results section should read as follows, with the corrections in bold:

**Variation in prevalence was evident between subsets of this population. Hispanics comprised a larger proportion of dual eligibles with MCC among those aged 65 or older compared with those aged 18 to 64, regardless of disability category. Alternately, non-Hispanic whites comprised a substantially larger proportion of those aged 18 to 64 than of those aged 65 or older. The proportion of women was higher than the proportion of men among those with MCC, regardless of age, but this differential was larger for those aged 65 or older. In education, twice the proportion of dual eligibles with MCC aged 65 or older had less than a high school education than seen among those aged 18 to 64. For dual eligibles with a high school education, there were higher proportions in the younger group than in the older group. **Dual eligibles aged 18 to 64 in each disability category had high obesity rates, as high as 60% among those with physical disabilities. High percentages of poor health status were reported by those aged 18 to 64, with the highest rate (39%) among dual eligibles with both physical disabilities and cognitive limitations; among those aged 65 or older, poor health status was highest among those with **both** physical disabilities and cognitive limitations. **A greater proportion of dual eligibles with MCC aged 65 or older lived in the South than in other regions of the country.**

This correction does not influence the accuracy of Table 2 or any other representation of the data elsewhere in the article, nor does it influence the discussion of the results.

The correction was made to our website on July 10, 2014, and appears online at http://www.cdc.gov/pcd/issues/2013/13_0064.htm. We regret any confusion or inconvenience this error may have caused.

